# Supplementary material for: Netrin-1 as a Multitarget Barrier Stabilizer in the Peripheral Nerve after Injury
Source: Int J Mol Sci. 2021 Sep 18;22(18):10090. doi: 10.3390/ijms221810090 (PMC8466625; doi:10.3390/ijms221810090)
Supplement: Supplementary file 1 [file ijms-22-10090-s001.zip › ijms-1355112-supplementary.pdf]

International Journal of
Molecular Sciences

MDPI

Supplementary Materials:

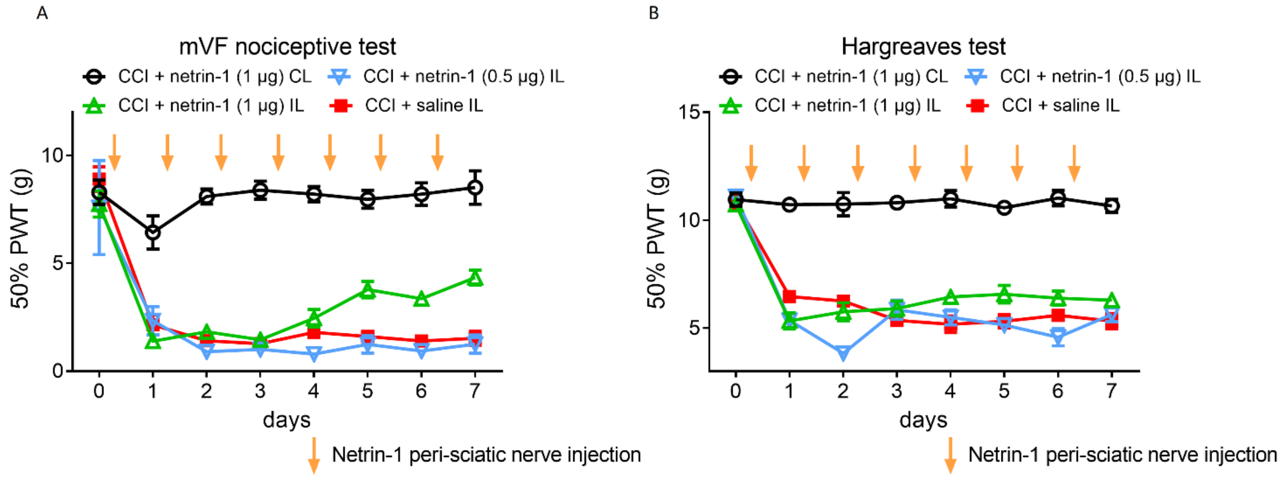

Figure S1. Antinociceptive effect of local netrin-1 application (1 or 0.5 µg) in CCI rats. Peri-sciatic nerve injections of 1 µg netrin-1 around the sciatic nerve slightly improved injury-induced hypersensitivity, but not 0.5 ug netrin-1. Starting on the day of surgery, recombinant netrin-1 injections in Wistar rats attenuated CCI-induced nociceptive hypersensitivity. Control rats were treated with solvents. (**A**) Mechanical nociceptive thresholds (as measured by von Frey filaments) and (**B**) thermal nociceptive thresholds (assayed by the Hargreaves' test) were measured 0 to 7 days after injury (*n* = 3–4/group). The orange arrows: netrin-1 peri-sciatic nerve injection.

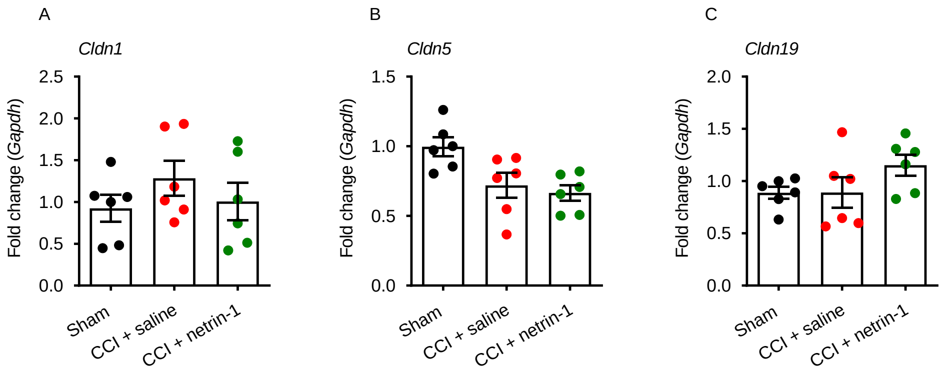

Figure S2. No changes in tight junction proteins in the dorsal root ganglion after systemic netrin-1 application. The tight junction protein *Cldn1* (**A**), *Cldn5* (**B**) and *Cldn19* (**C**) were quantified and analyzed in the dorsal root ganglions of sham and CCI treated rats (day 7) after daily systemic netrin-1 injection (10 µg). Gapdh was used as a housekeeping gene for the normalization of gene expression. All data are shown as mean ± SEM; *n* = 6/group.
